# Preparation, Characterization, and Antioxidant Activities of Extracts from *Amygdalus persica* L. Flowers

**DOI:** 10.3390/molecules29030633

**Published:** 2024-01-29

**Authors:** Qingtao Yu, Wenzhi Li, Ming Liang, Guohu Li, Zhuoyan Wu, Jieyi Long, Chanling Yuan, Wenjie Mei, Xiaole Xia

**Affiliations:** 1 Key Laboratory of Industrial Biotechnology, Ministry of Education, School of Biotechnology, Jiangnan University, Wuxi 214122, China; qingtao.yu@infinitus-int.com; 2 Infinitus (China) Company Ltd., Guangzhou 510405, China; peter.wenzhi.li@infinitus-int.com (W.L.); fiona.liang@infinitus-int.com (M.L.); anna.long@infinitus-int.com (J.L.); 3 Guangzhou Ruby Biotechnology Co., Ltd., Guangzhou 510006, China; liguohu0623@163.com (G.L.); ycc2552@163.com (C.Y.); 4 School of Pharmacy, Guangdong Pharmaceutical University, Guangzhou 510006, China; 18281834031@163.com; 5 Guangdong Province Engineering Technology Centre for Molecular Probes and Biomedicine Imaging, Guangzhou 510006, China; 6 College of Food Science and Engineering, Tianjin University of Science & Technology, Tianjin 300000, China

**Keywords:** *Amygdalus persica* L. flowers, polysaccharide, structural characterization, antioxidant activity

## Abstract

A novel water-soluble *Amygdalus persica* L. flowers polysaccharide (APL) was successfully isolated and purified from *Amygdalus persica* L. flowers by hot water extraction. Its chemical components and structure were analyzed by IR, GC-MS, and HPLC. APL consisted of rhamnose, arabinose, mannose and glucose in a molar ratio of 0.17:0.034:1.0:0.17 with an average molecular weight of approximately 208.53 kDa and 15.19 kDa. The antioxidant activity of APL was evaluated through radical scavenging assays using 1,1-diphenyl-2-picrylhydrazyl (DPPH), 3-ethylbenzthiazoline-6-sulfonic acid (ABTS), Hydroxyl radical scavenging, Superoxide radical scavenging, and the reducing power activity was also determined in vitro. Besides, in vivo antioxidant experiment, zebrafish (*Danio rerio*) embryos were treated with different concentrations of APL and then exposed to LPS to induce oxidative stress. Treatment with APL at 50 or 100 µg/mL significantly reduced LPS-induced oxidative stress in the zebrafish, demonstrating the strong antioxidant activity of APL. Moreover, the effect of APL on zebrafish depigmentation was tested by analyzing the tyrosinase activity and melanin content of zebrafish embryos. APL showed a potential reduction in the total melanin content and tyrosinase activity after treatment. This work provided important information for developing a potential natural antioxidant in the field of cosmetics and food.

## 1. Introduction

Oxidative stress has been pinpointed as a pivotal factor contributing to a variety of pathological conditions, including hypertension, cancer, cardiovascular and neurodegenerative diseases, and the aging process [1,2,3,4]. In recent years, evidence showed that excess free radicals produced metabolism in the body that can lead to oxidative stress [5]. This oxidative stress can lead to the oxidation and damage of vital macromolecules within organisms, ultimately disrupting signal transduction pathways and the equilibrium of antioxidants [6,7]. Elevated levels of intracellular reactive oxygen species (ROS) cause oxidative stress, leading to lipid, protein, and DNA damage [8]. It can damage cell structure and even cause cell death [9]. Studies have shown that DNA, lipids, and proteins in the brain tissue of patients with familial and sporadic PD are damaged by oxidative stress [10,11]. The effects of oxidative stress on the pathophysiology of cardiovascular diseases have also been reported to confirm that, in addition to causing cell dysfunction, necrosis, or apoptosis, ROS also induces specific protein post-translational modifications, thereby altering the function and signaling pathways of important cellular proteins in the heart [12,13,14,15]. Consequently, it is critical to seek natural compounds with potent antioxidant properties to prevent and treat these ailments.

It is reported that edible flowers are used as potential antioxidants to eliminate reactive oxygen species (ROS) produced during endogenous metabolic processes [16]. It is generally believed that polysaccharides in flowers exert antioxidant ability mainly through scavenging ability and reducing ability of different types of free radicals, and a few polysaccharides, such as Dendrobium officinale polysaccharide, also have high metal chelating activity. Therefore, polysaccharides in flowers can be used as a potential source of natural antioxidants. Polysaccharides, as abundant biomacromolecules in nature, exhibit a wide range of biological activities, attracting significant attention. It has been reported that plant polysaccharides from wild-growing plants like *Nymphaea hybrid*, *Lycopus lucidus*, and *Ganoderma lucidum*, demonstrate remarkable natural antioxidant properties without the toxicity associated with chemically synthesized antioxidants [17,18,19]. Furthermore, many plant polysaccharides possess anti-cancer, anti-inflammatory, and anti-aging properties, primarily attributed to their antioxidant effects [20]. Thus, plant polysaccharides are widely used as sustainable and renewable antioxidants in the food, cosmetic, and pharmaceutical industries.

Studies have found that edible flowers contain many antioxidants, and their extracts are beneficial to human health when eaten fresh and are a potential source of many natural antioxidants that can be used in the food and pharmaceutical industries [21,22,23]. It was also found that edible flowers may play an anticancer role by targeting oxidative stress and downstream dysregulation pathways [24]. *Amygdalus persica* L., a member of the Rosaceae family, is widely distributed across most regions of China and is known for its dual capacity to alleviate diarrhea and promote regular bowel movements while also inducing diuresis and alleviating edema [25]. *A. persica* flowers have a widespread application in traditional Chinese medicine and are also used as a healthy diet to enhance beauty and address constipation. Furthermore, they are utilized in the crafting of both wine and tea beverages. The health benefits of *A. persica* flowers are owing to thier various bioactive ingredients, such as polyphenols, flavonoids, polysaccharides, and other chemical components [26]. Zhang et al. isolated oleanolic acid, ursolic acid, quercetin, kaempferol, hesperetin, naringenin, and kaempferol-3-*O*-glucoside from *Amygdalus persica* L. flowers extract. Chen et al. found that the content of (+)-catechin in *Amygdalus persica* L. Flowers extract was 4588.15 μg/g DW, and the content of isoquercitrin was 1065.51 μg/g DW. These flavonoids and phenols may be related to the anticoagulant effects of *Amygdalus persica* L. Flowers extract [23,25]. Generally, polysaccharides are considered the main bioactive components, which possess various bioactivities, such as antioxidant, immunological, antitumor, hypoglycemic, and antimicrobial activities [27]. However, the polysaccharides from the *A. persica* flowers have not been systematically studied in their composition and antioxidants.

There are many methods for polysaccharide extraction, and hot water extraction is the most widely used method in polysaccharide extraction [28,29]. The principle is that most polysaccharides have a large solubility in hot water [30]. Polysaccharides are stable in hot water, so this extraction method has the least damage to polysaccharides. Some polysaccharides have higher extraction rates in lye [31]. In recent years, new technologies such as enzyme-assisted extraction [32], microwave-assisted extraction [33], ultrasonic-assisted extraction [34], and Supercritical fluid extraction [35] have continued to emerge In order to adapt to the extraction process of the vast production line, we chose the hot water extraction method to extract the polysaccharide.

In this work, we have extracted and characterized polysaccharides from *Amygdalus persica* L. flowers in hot water and evaluated their antioxidant activity in vitro and in vivo. First, the chemical composition, monomer units, and glycosidic bonds of the crude polysaccharides were investigated by spectroscopic and chromatographic analyses. The evaluation of the antioxidant activity of the crude polysaccharides demonstrated the synergistic relationship between the different components of the hot water-soluble extract and the type of glycoside bond to counteract radical and oxidant species. Thus, water-soluble polysaccharides are a source of natural antioxidants and can promote the consumption of the *Amygdalus persica* L. flowers as a functional food.

## 2. Result and Discussion

### 2.1. Isolation and Purification of Polysaccharide

**Figure 1:**
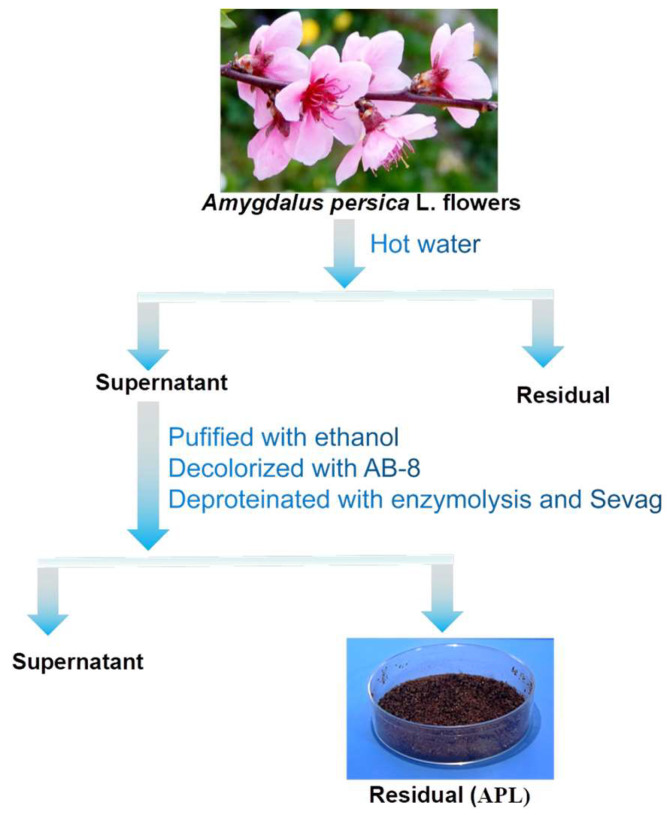
Isolation procedure of polysaccharides APL from the flowers of *Amygdalus persica* L.

Plant-derived natural polysaccharides are attracting increasing attention in the fields of food and healthy products, cosmetics, and so on due to their biological activities with non-toxic or low-toxic properties [36]. In this study, plant-derived crude polysaccharides were isolated from the flowers of *Amygdalus persica* L. with a yield of 11.2%. After the *Amygdalus persica* L. flowers were crushed, hot water was extracted and precipitated by ethanol; as shown in Figure 1, methods for removing pigments and proteins from crude polysaccharides were used by novel, green, and efficient technologies. As reported, the anion exchange macroporous resin method is milder, simpler, and more effective compared with methods of H_2_O_2_ oxidation decolorization [37]. Thus, we used the AB-8 macroporous resin for the purpose of depigmentation. Sevag method is effective and widely used to remove free protein. However, the major problem of this approach is that the Sevag method is low-efficiency and tends to cause the loss of polysaccharides. In order to ensure a complete protein removal, we used enzymolysis combined with the Sevag method to remove free protein. Repeat until there is no significant precipitation.

### 2.2. Characterization of APL

**Figure 2:**
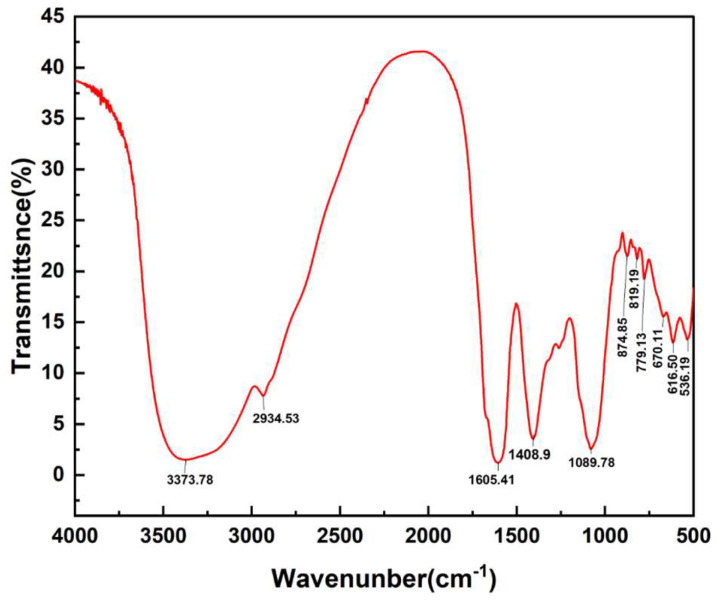
FT-IR spectrum of APL from the flowers of *Amygdalus persica* L.

The FT-IR spectroscopy is widely utilized to provide insights into the distinctive groups present in polysaccharides. The FT-IR results displayed in Figure 2 exhibit the typical absorption peaks of APL. As shown in Figure 2, there was a broad and strong absorption peak of polysaccharides at 3373.78 cm^−1^, which can be attributed to hydroxyl -OH groups. Peaks at 2934.53 cm^−1^ and 1408.9 cm^−1^ were ascribed to the stretching vibration of C-H bonds. The band at approximately 1605.41 cm^−1^ was attributed to the stretching vibration of C=O. Peaks detected at 1089.78 cm^−1^ could be attributed to the contribution of C-O-C symmetric stretching vibration. Absorption peaks at 874.85 cm^−1^ suggested the presence of β-configurations within APL’s molecular structure. The FTIR spectra exhibited all typical absorption peaks associated with polysaccharides, which confirmed the identity of APL as a polysaccharide [38].

**Figure 3:**
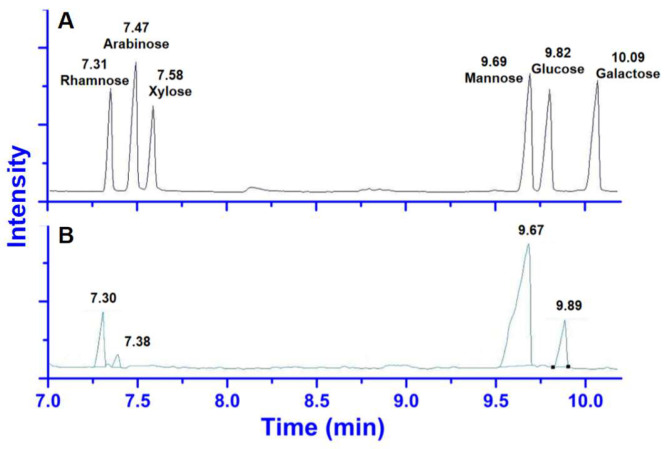
The GC traces of monosaccharide standards (**A**) and APL (**B**).

Compared with the derivatives of monosaccharide standards, the GC trace of the trifluoroacetic acid derivatives of the hydrolyzed APL (Figure 3) indicated that APL was predominantly composed of rhamnose, arabinose, mannose, and glucose in a molar ratio of 0.17:0.034:1.0:0.17. Mannose was the main monosaccharide.

Since molecular weight affects the configuration and morphology of polysaccharides, its determination is crucial in the study of polysaccharides. According to the calibration curve of the dextran standards (y = −0.339x + 9.1997, R^2^ = 0.9918), as shown in Table 1, the average molecular weight of the APL was calculated to be approximately 208.53 kDa and 15.19 kDa.

### 2.3. In Vitro Antioxidant Activity Analysis

#### 2.3.1. Scavenging Activity of DPPH Radicals

**Figure 4:**
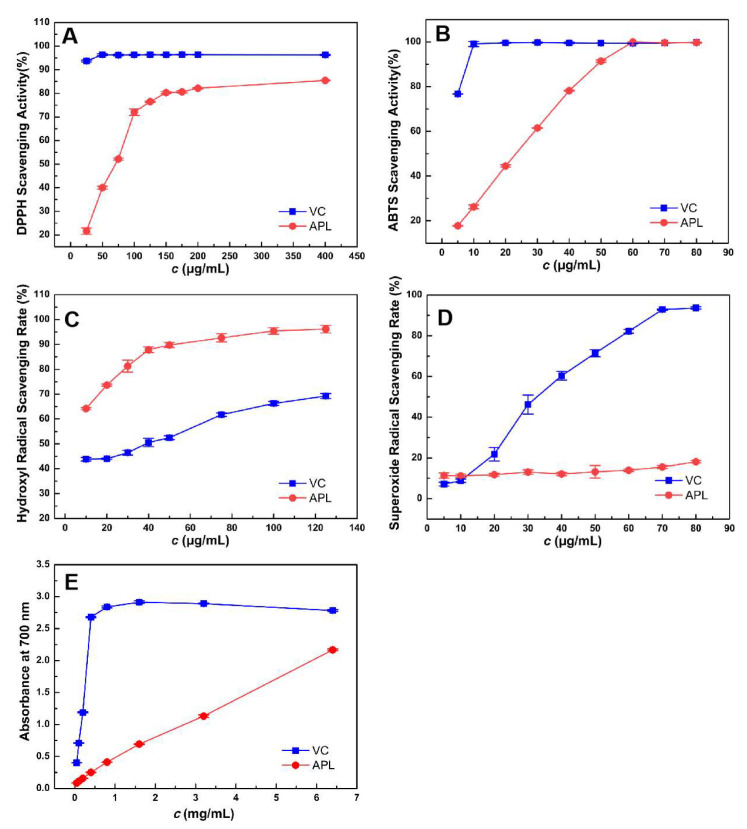
In vitro antioxidant activity of APL. (**A**) DPPH radical scavenging assay, (**B**) ABTS•+ radical scavenging assay, (**C**) Hydroxyl radical scavenging assay, (**D**) Superoxide radical scavenging assay, (**E**) the reducing power ability. Values are presented as mean ± SD of triplicate experiments.

The scavenging activity of DPPH, a stable free radical, was widely indexed and a quick method to measure the free radical-scavenging activities of antioxidants with hydrogen molecule conation [39]. Since the polysaccharides can function as electron donors and interact with free radicals, thereby transforming them into more stable products, they possess the capacity to halt radical chain reactions. As shown in Figure 4A, when the concentration of APL is less than 100 µg/mL, it is obvious from the results that with the increase of APL concentration, the scavenging effect of DPPH radicals becomes more obvious, almost showing exponential growth, and there is a significant concentration-dependent effect. When the concentration of APL was between 100–200 µg/mL, the scavenging effect of APL on DPPH free radicals tended to be flat with the increase in sample concentration. At the concentration of 400 µg/mL, the scavenging ability of APL reached the maximum value (85.45 ± 0.12%). Our results indicated that APL was effective in scavenging DPPH radicals.

#### 2.3.2. Scavenging Activity of ABTS Radicals

ABTS method is used to evaluate the total antioxidant capacity [40]. The variation trend of scavenging activity of APL against ABTS radicals was similar as that against DPPH radicals. As shown in Figure 4B, with the increase of the concentration of VC and APL, the ability to scavenge ABTS free radicals increased gradually, and VC showed a strong scavenging ability of ABTS free radicals at low concentrations. With the increase of APL, the scavenging ability was significantly enhanced. Nobly, at the concentration of 60 µg/mL, the scavenging ability of APL was 99.96 ± 0.066%, a little higher than VC (99.39 ± 0.22%).

#### 2.3.3. Hydroxyl Radical Scavenging Activity

Hydroxyl radicals are reactive oxygen species (ROS) formed during the metabolic process in living organisms [41]. Upon entry into a cell, hydroxyl radicals readily react with various biological molecules, leading to imbalances and damage within the organism. Hence, the removal of hydroxyl radicals holds immense importance for human health. The scavenging activities of APL and VC against hydroxyl radicals are shown in Figure 4C. The scavenging activity of APL exhibited a significant increase from 64.15 ± 0.42% to 89.74 ± 0.98% as the concentration ranged from 10 to 50 µg/mL. When the concentration exceeded 50 µg/mL, the scavenging activity increased gradually, with a trend towards complete elimination of hydroxyl radicals. It is worth noting that within the concentration range of 10 to 125 µg/mL, the scavenging capacity of APL exceeded that of VC, indicating that APL possesses stronger scavenging capabilities.

#### 2.3.4. Superoxide Radical Scavenging Activity

Superoxide anion radicals (O^2•−^) possess an extended lifespan compared to other radicals, even though they are less reactive. They can engage in additional reactions, contributing to the generation of various reactive oxygen species. When present in excess, these species can disrupt the organism’s equilibrium, promoting DNA damage and the proliferation of various diseases [42]. As shown is Figure 4D, under the experimental conditions, the activity of scavenging superoxide is better with the increase of VC concentration. With the increase of APL concentration, the inhibitory effect on superoxide did not change much. The superoxide radical scavenging activity rate of APL was 18.13 ± 0.59% at the concentration of 80 µg/mL, exhibiting a weak superoxide radical scavenging activity.

#### 2.3.5. Reducing Power

The potential antioxidant activity of specific bioactive substances can be effectively demonstrated through the reducing power assay, which involves the reduction of oxidized intermediates [43]. In this study, the capacity of polysaccharides APL from *Amygdalus persica* L. flowers to facilitate the reduction of Fe^3+^ to Fe^2+^ was measured. As shown in Figure 4E, when VC is lower than 1.0 mg/mL, the reduction capacity is significantly improved with the increase of VC concentration. When VC is greater than 1.0 mg/mL, the reduction capacity tends to be stable and has a slightly decreasing trend. However, with the increase of APL concentration, the reducing ability was gradually enhanced. At the concentration of 6.4 mg/mL, APL exhibited an absorbance of 2.17, while VC had an absorbance of 2.78, and the reduction capacity of the two is not different at the same concentration, suggesting that APL possessed robust antioxidant capabilities and could serve as a potential functional food additive for the prevention or alleviation of oxidative stress.

### 2.4. Validation of Antioxidant Activity In Vivo

#### 2.4.1. Effect of APL on Fluorescence Intensity of ROS in Zebrafish

**Figure 5:**
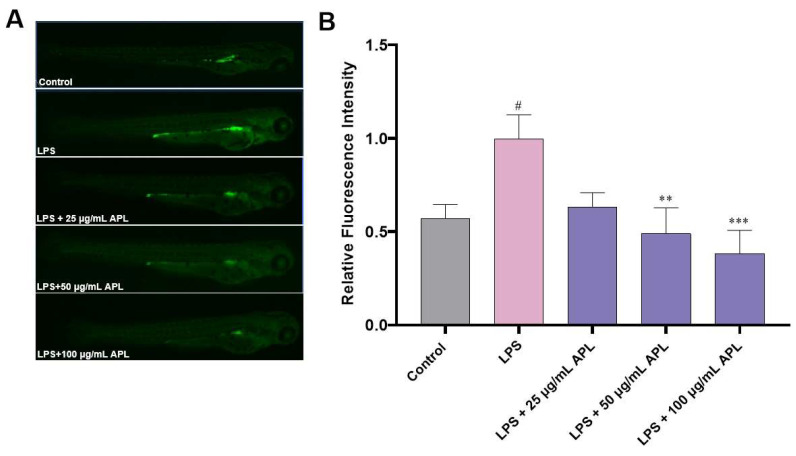
Inhibitory effect of APL on LPS-induced ROS in zebrafish. (**A**) ROS fluorescence images of zebrafish treated with different concentrations of APL. (**B**) Quantification of fluorescence intensity by Image J (v1.48 software). The bars represent ± SEM. **^#^** *p* < 0.05, when compared with the control. ** *p* < 0.01, *** *p* < 0.001, when compared with the model group.

Detecting ROS production is crucial for understanding various biological processes, identifying disease mechanisms, assessing environmental risks, and developing therapeutic strategies [44]. It provides valuable information across multiple scientific disciplines and has significant implications for human health, environmental protection, and technological advancements. The oxidative stress model can be constructed using LPS induction [45]. In this section, the oxidative stress of zebrafish was induced using LPS, and the ROS content in Zebrafish was detected using a fluorescence probe DCFH-DA. The effect of APL on LPS-stimulated cytokine reactive oxygen species is shown in Figure 5. LPS model group (1.00 ± 0.13) was higher than that in the normal control group (0.57 ± 0.08), *p* < 0.05, indicating the successful establishment of the oxidative stress model. After 25 µg/mL APL treatment, the relative fluorescence intensity of ROS in zebrafish was 0.63 ± 0.08, which decreased by about 37% compared with the model group. There was no significant difference between the model group and the 25 µg/mL con-centration sample group through one-way ANOVA. In contrast, after exposure to the higher-concentration APL treatment groups (50 and 100 µg/mL), ROS levels were significantly enhanced with increasing the concentration of APL. At a concentration of 50 µg/mL, the relative fluorescence intensity of ROS was 0.49 ± 0.14. When the concentration of APL was increased to 100 µg/mL, the relative fluorescence intensity of ROS was 0.38 ± 0.12, which was 62% lower than that of the model group. Therefore, these findings suggest that APL exhibits effective antioxidant properties.

#### 2.4.2. Effect of APL on Tyrosinase Activity and Melanin Distribution

The zebrafish is now a favored model for biochemical research, providing an effective alternative to animal experiments, and studying surface pigmentation in zebrafish offers a simple approach to investigating pigmentation processes without complex lab procedures [46]. Tyrosinase is a copper-containing metal oxidoreductase, widely distributed in animals, plants, microorganisms, and the human body, with monophenolase and diphenolase activities. The dinuclear copper ion in its active center plays an important role in enzyme catalysis and is a key enzyme in the production of melanin. Inhibiting its activity can effectively prevent the production of melanin. According to the different inhibition mechanisms of tyrosinase inhibitors, it can be divided into destructive inhibition of tyrosinase and non-destructive inhibition of tyrosinase. Destructive inhibition of tyrosinase, also known as irreversible inhibition, refers to the destruction of the active site of tyrosinase so that inhibitor molecules and tyrosinase irreversible binding or damage to the structure of the enzyme, and finally cause tyrosinase to lose physiological function. At present, this type of inhibitor is mainly limited to the destruction of the active site of tyrosinase, such as Cu^2+^, because tyrosinase is a binding enzyme, and copper ion acts as a cofactor, and the activity of tyrosinase will be significantly decreased when the content of copper ion is reduced. Sulfhydryl compounds in the human epidermis, especially glutathione, can inhibit tyrosinase activity by complexing copper ions. The non-destructive inhibition of tyrosinase is also called reversible inhibition; that is, without modification or modification of tyrosinase itself, the combination of inhibitor molecules and tyrosinase is reversible, and the purpose of inhibiting melanin formation is achieved by inhibiting the biosynthesis of tyrosinase or replacing the substrate of tyrosinase. The non-destructive inhibition mechanism of tyrosinase can be divided into four types: competitive inhibition, non-competitive inhibition, anti-competitive inhibition, and mixed (competitive/non-competitive) inhibition. For example, inhibition of tyrosinase using Quercetin is reversibly competitive [47]. Kojic acid can competitively inhibit the activity of monophenolase tyrosinase [48]. Tricin is a non-competitive inhibitor of tyrosinase [49]. Choi J and Jee JG found that thiourea-containing drugs were non-competitive tyrosinase inhibitors, while the reference molecules (PTU and kojic acid) were classified as competitive inhibitors [50].

**Figure 6:**
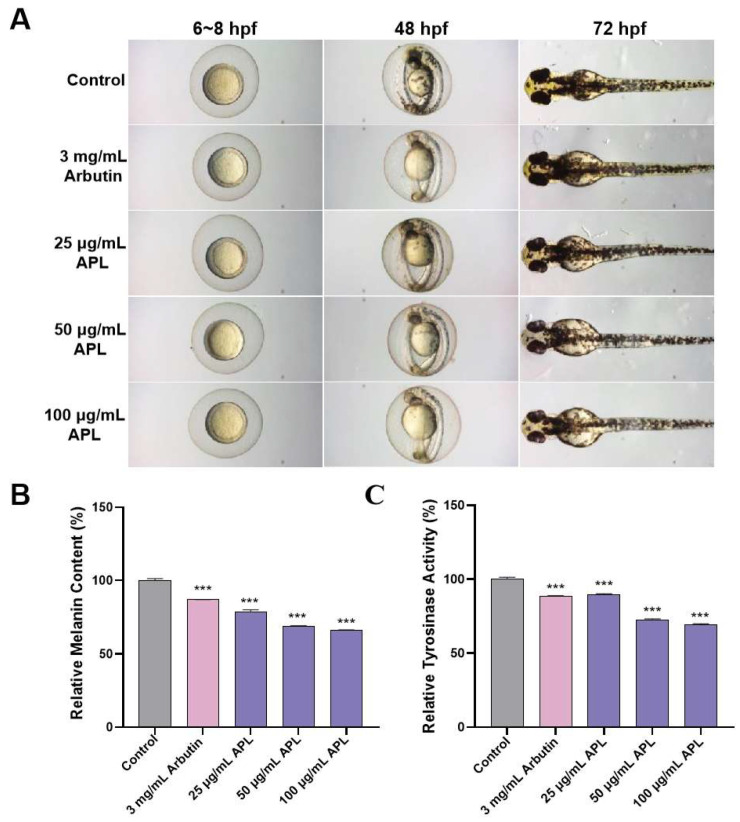
Effects of APL on the pigmentation in zebrafish. (**A**) Representative photographs of zebrafish after treatment with various concentrations of APL for 6~8 hpf, 48 hpf and 72 hpf. Arbutin was used as a positive group. (**B**) Relative melanin content and (**C**) relative tyrosinase activity were determined after treatment with various concentrations of APL for 72 hpf. The bars represent ± SEM. *** *p* < 0.001 when compared with the control.

Arbutin, chemical formula C_12_H_16_O_7_, is a component extracted from the azalea plant arbutin leaves. It can inhibit the activity of tyrosinase in the body and prevent the production of melanin, thereby reducing skin pigmentation, removing stains and freckles, as well as bactericidal and anti-inflammatory effects. Therefore, arbutin is used as an effective raw material for freckle-whitening cosmetics. In order to study the inhibitory activity of APL on tyrosinase, we used arbutin as a positive control. The evident whitening effect of APL on the embryos at 25 µg/mL, 50 µg/mL, and 100 µg/mL is also supported by the images shown in Figure 6A. In the negative control group, zebrafish embryos exhibited surface melanin at 48 hpf, which became more pronounced at 72 hpf. The positive group (arbutin, 3 mg/mL) showed a reduction in the number of black spots compared to the control group, and the relative melanin content and relative tyrosinase activity were 86.85 ± 0.17% and 88.63 ± 0.22%, respectively, with significant differences compared with the blank group (*** *p* < 0.001), indicating its inhibitory effect on black spot formation. Compared with the control group, the number of black spots on the zebrafish embryo treated with APL gradually decreased in a dose-dependent manner. Furthermore, the result of quantitative analysis on zebrafish depigmentation was tested by analyzing the tyrosinase activity and melanin content of zebrafish embryos 72 h after treatment. With the increase in APL concentration, the relative contents of melanin in zebrafish treated with APL were 89.67 ± 0.22%, 72.66 ± 0.59%, and 66.12 ± 0.29%, respectively. The relative tyrosinase activities of zebrafish treated with different concentrations of APL to 72 hpf were 78.58 ± 1.39%, 68.71 ± 0.55%, and 69.52 ± 0.43%, respectively. The Figure 6B,C shows a potential reduction in the total melanin content and tyrosinase activity after treatment. These results indicated that APL was as effective as arbutin in inhibiting zebrafish pigmentation. The concentration was lower than that of arbutin.

## 3. Materials and Methods

### 3.1. Materials

The flowers of *Amygdalus persica* L. were cultivated in Pingyi County, Linyi City, Shandong Province of China. AB-8 and D101 macroporous resin was obtained from Cangzhou Bon Adsorber Technology Co., Ltd. (Cangzhou, China). 1,1-diphenyl-2-picrylhydrazyl (DPPH), L-Ascorbic acid, 2,2’-azino-bis (3-ethylbenzothiazoline-6-sulfonic acid) (ABTS), arbutin, deoxycholic acid sodium salt, 3,4-Dihydroxy-L-phenylalanine were procured from Shanghai Macklin Biochemical Co., Ltd. (Shanghai, China). Standard monosaccharides (including arabinose, rhamnose, xylose, fructose, mannose, galactose and glucose), lipopolysaccharides (LPS) and 2’,7’-Dichlorofluorescein diacetate (DCFH-DA) were purchased from Sigma-Aldrich (St. Louis, MO, USA). All reagents and chemicals were of analytical grade unless stated otherwise. Distilled water was used in all experiments.

### 3.2. Exaction and Purification of Polysaccharides

The crushed and dried flowers of *Amygdalus persica* L., as shown in Figure 1, were purchased from a market in China. The obtained sample was mixed with distilled water in a Soxhlet extractor, following a solvent-to-raw material ratio of 20:1. Subsequently, extraction was conducted at 100 °C for 1 h. This extraction procedure was repeated twice. The suspension was centrifuged (4500 rpm, 10 min) and concentrated to 30% of the initial volume using a rotary evaporator under reduced pressure. The above supernatant was further purified by precipitating with 95% ethanol/water and left at 4 °C overnight. The precipitate was collected by centrifugation at 4500 rpm for 10 min. Dissolve the obtained precipitate in pure water using a 5:1 weight ratio. Utilize a mixture of AB-8 and D101 macroporous resins at a 1:1 ratio for the decolorization process. Protein removal was accomplished using the Enzymolysis and Sevag method. The polysaccharide solution was added with papain (enzyme activity 100,000 U/g), the enzyme was put in a 60 °C water bath for 1 h, and then the enzyme was inactivated in a boiling water bath for 10 min. The sample solution was removed and the volume ratio of polysaccharide solution to Sevag reagent was 1:1. Finally, the resulting solution was lyophilized to obtain the crude polysaccharides, coded as APL, which exhibited a brown powder appearance, as shown in Figure 1. The contents of total sugar were measured using the phenol-sulfuric acid method [51].

### 3.3. Analysis of Monosaccharide Components

The sample APL was hydrolyzed using 2 M trifluoroacetic acid (TFA) at 100 °C for 6 h. Following the elimination of excess TFA, the hydrolyzed products of the polysaccharide were subjected to silyl etherification using 0.3 mL of TriSil reagent, with a mixture of pyridine, hexamethyldisilazane, and trimethylchlorosilane in a ratio of 10:2:1. Subsequently, the hydrolysates were diluted with distilled water and measured through an ion chromatography (TSQ 8000 Evo GC-MS/MS, Thermo Fisher, Waltham, MA, USA). The same method was applied for the analysis of monosaccharide standards (including arabinose, rhamnose, xylose, fructose, mannose, galactose, and glucose) with varying concentrations.

### 3.4. Determination of Molecular Weight Distribution

The homogeneity and average molecular weight of APL were determined by high-performance gel permeation chromatography (HPGPC) with a TSK gel G-4000 PWxL column (7.8 mm × 300 mm, Tosoh Biosep, Chonan, Japan). 20 μL APL was injected in each run at room temperature and was eluted with ultrapure water at a flow rate of 0.6 mL/min. The temperature of the column was maintained at 30 °C. Column calibration was conducted using the standard dextrans with different *M*_w_ (10,000 Da, 39,994 Da, 69,984 Da, 109,900 Da, and 1,999,862 Da). The molecular weights were estimated by referencing the calibration curve made under the previously mentioned conditions, using the standard dextran of known molecular weights as reference points.

### 3.5. FT-IR Spectroscopic Analysis

The Fourier transform-infrared (FT-IR) spectra of APL in the range of 4000–400 cm^−1^ were recorded using the Nicolet™ iS5 FTIR spectrometer (Thermo Electron, Madison, WI, USA) through the KBr tablet pressing method.

### 3.6. In Vitro Assays of Antioxidant Activity

#### 3.6.1. DPPH Radical Scavenging Activity

DPPH radical scavenging activity was investigated following a previously reported method with some modifications. In brief, DPPH radicals were prepared by dissolving in anhydrous ethanol (0.11 mM), while the various concentrations of polysaccharide solutions were prepared by dissolving in ultrapure water. We mixed 100 µL of DPPH solutions and 100 µL of each sample, then incubated them at 25 °C for 35 min in the dark. Finally, the absorbance was measured at 517 nm. The reaction mixtures without DPPH were used as a control, and L-Ascorbic acid (VC) was used for positive control. The scavenging activity SA_DPPH_ was calculated according to the following Equation (1):

(1) $$SA=\left(1-\frac{A_{1}-A_{2}}{A_{0}}\right)\times 100$$

Here, *SA* is the scavenging activity of the tested samples (%), *A*_1_ is the absorbance of the reactant solution, *A*_2_ is the absorbance the sample solution, and *A*_0_ is the absorbance of the blank solution.

#### 3.6.2. ABTS Radical Scavenging Activity

ABTS radical scavenging activity was measured using a modified method [52]. The ABTS reaction solution was prepared using the following steps. First, equal volumes of 7 mM ABTS and 2.45 mM K_2_S_2_O_8_ solution were mixed and reacted in the dark for 12~16 h. Subsequently, the solution was diluted with 5 mM phosphate buffer (PBS, pH 7.4) to obtain an absorbance of approximately 0.70 at 734 nm. For the experiment, the samples, positive control, and blank group were mixed with the ABTS working solution at a ratio of 1:4, respectively. The mixtures were then incubated in darkness at room temperature for 10 min before measuring the absorbance at 734 nm. PBS was used as a control, and VC was used as a positive control. The scavenging activity SA_ABTS_ was calculated according to Equation (1).

#### 3.6.3. Hydroxyl Radical Scavenging Activity

Hydroxyl radical scavenging activity was executed using the Fenton method [53] with a minor amendment. In brief, 0.2 mL of sample solutions of different concentrations, 1 mL of FeSO_4_ solution (0.15 mM), 0.4 mL of H_2_O_2_ solution (2 mM), 0.4 mL of distilled water, and 1 mL of salicylic acid ethanol solution (6 mM) were mixed thoroughly, then incubated at 37 °C for 1 h. The absorbance was measured at 510 nm. Distilled water was used as a control, and VC was taken as a positive control. The hydroxyl radical scavenging activity SA_OH_ was calculated according to Equation (1).

#### 3.6.4. Superoxide Radical Scavenging Activity

Superoxide radical scavenging activity was measured according to the previous method with some modifications [54]. The system that generates superoxide radicals was based on the autoxidation of the pyrogallol reaction. 0.125 mL of sample solutions of different concentrations were mixed with 1.125 mL of Tris-HCl buffer (50 mM, pH 8.0). After incubating for 20 min at 25 °C, 0.25 mL of pyrogallol solution (25 mM), which had been preheated, was added to the mixture, and the mixture was incubated for 5 min at 25 °C. Then 0.25 mL of HCl solution (8 mM) was added to terminate the reaction, and the absorbance was measured at 320 nm. Tris-HCl buffer was used as a control, and VC was taken as a positive control. The superoxide radical scavenging activity SAo_2_^−^ was calculated according to Equation (1).

#### 3.6.5. Reducing Power

The reducing power was measured using K_3_Fe(CN)_6_ according to the established method [55]. A 1 mL measure of different concentrations of sample solution, 2.5 mL of 0.1% K_3_Fe(CN)_6_ (*w*/*v*) solution, and 2.5 mL of 0.2 M phosphate buffer solution (pH 6.6) were homogeneously mixed and reacted at 50 °C for 20 min. We added 2 mL of 10% TCA (*w*/*v*) to the mixture, then centrifuged it at 3000 r/min for 10 min. We added 2 mL of distilled water and 0.5 mL of 0.1% FeCl_3_ (*w*/*v*) to 2 mL of supernatant, mixed well, and reacted at 50 °C for 10 min. The absorbance of samples was determined at 700 nm. VC was used as a positive control. The reduction force is calculated according to Equation (2)

(2) $$Reducing\;power=A_{1}-A_{2}$$

where *A*_1_ is the absorbance of reactant solution, *A*_2_ is replaced with the FeCl_3_ solution with distilled water.

### 3.7. In Vivo Assays of Antioxidant Activity

#### 3.7.1. Zebrafish Culture

The wild-type AB zebrafish (*Danio rerio*) were purchased from the China Zebrafish Resource Center (CZRC, Wuhan, China). The zebrafish were raised and maintained in a recirculating aquaculture system at a constant temperature of 28 ± 1 °C under a 14 h light/10 h dark cycle, and they were fed brine shrimp three times a day. Zebrafish embryos were obtained through natural pair-wise mating with a 1:1 sex ratio. Within 1.5 h after the light was switched on, the embryos were collected, washed with circulating water, transferred into clean Petri dishes, and maintained with embryo medium in a pathogen-free environment with ad lib feeding. The procedures for the care and use of animals were approved by the Ethics Committee of Guangdong Pharmaceutical University. All animal experiments were conducted in compliance with the ARRIVE guidelines and carried out in accordance with the National Institutes of Health Guide for the Care and Use of Laboratory Animals.

#### 3.7.2. Assay of Antioxidant Effect in Zebrafish

The production of ROS in zebrafish embryos was assessed using an oxidation-sensitive fluorescent probe dye, 2,7-dichlorofluorescein diacetate (DCFH-DA) [56]. Wild-type zebrafish embryos (6~9 hpf) were randomly selected and transferred to a 6-well plate with 20 embryos per well containing 4.5 mL embryo medium. The zebrafish embryos were treated with APL (0, 25, 50, and 100 µg/mL), and 40 min later, 10 µg/mL lipopolysaccharide (LPS) was added to the plate. After treatment with 10 µg/mL LPS for 24 h, the embryo medium was changed, and the embryos developed up to 72 hpf. The zebrafish embryos were incubated with DCFH-DA (20.0 μg/mL) for 1 h in the dark at 28 °C. After incubation, the embryos were rinsed 2–3 times with aquaculture water, followed by anesthetized with 0.02% tricaine. Subsequently, they were observed and recorded under a fluorescence microscope (Leica DMI8). The fluorescence intensity was quantified and analyzed using Image J v1.48 software.

#### 3.7.3. Assay of Depigmentation Effect and Tyrosinase Activity in Zebrafish

After 72 h exposure, 30 embryos were collected and sonicated in a lysis buffer containing 5 mg/mL deoxycholic acid sodium salt and then clarified by centrifugation at 12,000 rpm for 5 min at 4 °C. The supernatant was utilized for measuring tyrosinase activity, while the pellet was employed to ascertain melanin content. The pellet was dissolved in 150 μL of 1 M NaOH solution, and the absorbance was read at 405 nm to determine the melanin content. To assess the inhibition of tyrosinase activity, 100 μL of the supernatant was combined with 100 μL of 0.95 mg/mL solution of 3,4-Dihydroxy-L-phenylalanine. The mixture was then incubated at 37 °C for 1 h, and absorbance was measured at a wavelength of 475 nm. Tyrosinase activity = (1 − ODa/ODb) × 100%, ODa, and ODb were the absorbance values of the experimental groups and control groups, respectively. The melanin contents and specific tyrosinase activity were expressed as the percentage change in comparison to that in the control group, which was considered to be 100%. The embryos treated with arbutin were used as the positive control.

### 3.8. Statistical Analysis

The results were expressed as the mean ± standard deviation (SD) or mean ± mean standard error (SEM) in triplicates. Statistical analysis was performed using GraphPad Prism 8.0.2 software and analyzed using one-way ANOVA analysis. Differences with * *p* < 0.05, ** *p* < 0.01, or *** *p* < 0.001 were considered statistically significant.

## 4. Conclusions

Polysaccharides derived from plants are one of the important natural antioxidants. In this study, plant-derived polysaccharides were isolated from the flowers of *Amygdalus persica* L. using hot water exaction. This was the first report to explore the chemical characterization and antioxidant activities of polysaccharide extracts from *Amygdalus persica* L. flowers. A novel polysaccharide fraction APL consisted of rhamnose, arabinose, mannose, and glucose in a molar ratio of 0.17:0.034:1.0:0.17 with an average molecular weight of approximately 208.53 kDa and 15.19 kDa. The polysaccharide APL exhibited outstanding radical scavenging activities and reduced power capacity in vitro antioxidant activity assays. Moreover, APL can reduce the production of ROS in a LPS-induced oxidative stress model in zebrafish. The whitening effect assessed in zebrafish proved that APL can inhibit melanin production. Overall, APL could be developed as an active ingredient with natural antioxidant properties, including whitening and protection against oxidative damage, which promotes a prospect as a functional raw material in the field of cosmetics and food industries. Due to the simple in vivo experimental verification, the antioxidative and inhibiting tyrosinase activity mechanism of APL needs to be further studied, and the separation and purification methods need to be further optimized.

## Figures and Tables

**Table 1:** Characteristics of APL purified from the flowers of *Amygdalus persica* L.

Items	Amount
Rhamnose (molar ratio)	0.17
Arabinose (molar ratio)	0.034
Mannose (molar ratio)	1.0
Glucose (molar ratio)	0.17
Weight-average molecular weight (*M*_w_, kDa)	208.53 (56.2%)
Weight-average molecular weight (*M*_w_, kDa)	15.19 (33.3%)

## Data Availability

Data are contained within the article.
